# Heme oxygenase-1 is critically involved in placentation, spiral artery remodeling, and blood pressure regulation during murine pregnancy

**DOI:** 10.3389/fphar.2014.00291

**Published:** 2015-01-13

**Authors:** Maria L. Zenclussen, Nadja Linzke, Anne Schumacher, Stefan Fest, Nicole Meyer, Pablo A. Casalis, Ana C. Zenclussen

**Affiliations:** Experimental Obstetrics and Gynecology, Medical Faculty, Otto-von-Guericke University Magdeburg, Magdeburg, Germany

**Keywords:** heme oxygenase-1, pregnancy, implantation, placenta, pre-eclampsia, IUGR

## Abstract

The onset of pregnancy implies the appearance of a new organ, the placenta. One main function of the placenta is to supply oxygen to the fetus via hemoproteins. In this review, we highlight the importance of the enzyme heme oxygenase-1 (HO-1) for pregnancy to be established and maintained. HO-1 expression is pivotal to promote placental function and fetal development, thus determining the success of pregnancy. The deletion of the gene *Hmox1* in mice leads to inadequate remodeling of spiral arteries and suboptimal placentation followed by intrauterine growth restriction (IUGR) and fetal lethality. A partial *Hmox1* deletion leads to IUGR as well, with heterozygote and wild-type fetuses being born, but *Hmox1*^–/–^ significantly below the expected Mendelian rate. This strong phenotype is associated with diminished number of pregnancy-protective uterine natural killer (uNK) cells. Pregnant heterozygote females develop gestational hypertension. The protective HO-1 effects on placentation and fetal growth can be mimicked by the exogenous administration of carbon monoxide (CO), a product of heme catalyzed by HO-1. CO application promotes the *in situ* proliferation of uNK cells, restores placentation and fetal growth, while normalizing blood pressure. Similarly, HO-1 inhibition provokes hypertension in pregnant rats. The HO-1/CO axis plays a pivotal role in sustaining pregnancy and aids in the understanding of the biology of pregnancy and reveals a promising therapeutic application in the treatment of pregnancy complications.

## INTRODUCTION

Heme oxygenase-1 (HO-1) catalyzes the first and rate-limiting step in heme catabolism toward biliverdin, carbon monoxide (CO), and free iron (Tenhunen et al., 1968). HO-1 represents the stress-responsive HO isoform and is encoded by the *Hmox1* gene. As shown *in vitro* and *in vivo* studies, HO-1 is cytoprotective and exerts anti-inflammatory effects, while regulating cell proliferation (Soares et al., 1998; Otterbein et al., 2000; Duckers et al., 2001; Soares and Bach, 2009). HO-1 not only prevents tissue injury; but also, modulates innate and adaptive immune responses (Duckers et al., 2001). Thus, HO-1 is a central player in suppressing the pathogenesis of immune-mediated inflammatory diseases (Soares et al., 2009). The cytoprotective and immunoregulatory effects of HO-1 no longer exist after the pharmacological inhibition of its enzymatic activity. The exogenous application of CO by inhalation is able to restore the cytoprotective effects HO-1 (Brouard et al., 2000; Otterbein et al., 2000; Ryter et al., 2002). Hence, CO mediates, to a large extent, the salutary effects of HO-1. However, other products of heme catabolism such as iron and biliverdin, might act in a similar manner. In this review, we will focus on the protective effects of HO-1 and its main metabolite CO.

## HO-1 EXPRESSION IN FEMALE REPRODUCTIVE ORGANS

Alexandreanu and Lawson (2003) reported that rat theca cells, granulosa cells of follicles, and luteal cells stain positively for HO-1; whereas, ovarian stromal cells have a low intensity of staining. They further showed that three daily injections of the HO-1 inducer hemin stimulated basal- and gonadotropin-induced androstenedione and estradiol secretion from ovaries of immature rats treated with pregnant mare serum gonadotropin *in vitro*, but had no effect on progesterone production (Alexandreanu and Lawson, 2003). Thus, HO-1 is present in the rat ovary and may be involved in the production of ovarian steroids. In mice, HO-1 is expressed in the ovaries and HO-1-deficient mice have deficient ovulation (Zenclussen et al., 2012). Contrary to the data obtained in the rat, variations in HO-1 did not influence the production of female hormones in the mouse (Zenclussen et al., 2011). However, estrous cycle-related changes in hormonal levels significantly modified the temporal expression of HO-1 in uterine cells (Zenclussen et al., 2014).

Upon fecundation, HO-1 is expressed very early in pregnancy. Already at the blastocyst stage, its presence determines the attaching ability of blastocysts to uterine epithelial cells (UECs; Zenclussen et al., 2011). As this stage is determinant for the implantation success, the absence of HO-1 implies a deficient or absence of implantation.

HO-1 is expressed in trophoblasts as shown in human studies (Acevedo and Ahmed, 1998; Ahmed et al., 2000; Lyall et al., 2000; McLean et al., 2000; Zenclussen et al., 2003), mice (Zenclussen et al., 2005, 2006; Zhang et al., 2007; Tachibana et al., 2008) and rat (Ihara et al., 1998; Kreiser et al., 2002) placentas. There is a consensus that HO-1 expression in trophoblasts is indicative of adequate placental development and fetal well-being. It is highly induced during human, rat, and mouse pregnancies, namely in placental trophoblasts (Barber et al., 2001; Zenclussen et al., 2003; Sollwedel et al., 2005). HO-1 induction supports pregnancy, diminishing the early onset of murine abortions (Sollwedel et al., 2005; Zenclussen et al., 2006). Reduced HO-1 levels are associated with human miscarriages and murine fetal losses (Zenclussen et al., 2003, 2005; Sollwedel et al., 2005) and pre-eclampsia, the most severe pathological complication of pregnancy (Ahmed et al., 2000).

Due to its continual presence at all stages of the reproductive cycle and during pregnancy, HO-1 is believed to play a central regulatory role in reproduction. In fact, there are limited examples of genes whose expression is strictly required at multiple stages of pregnancy in mammals (Wang and Dey, 2006).

Very few *Hmox1* null (*Hmox1*^–/–^) mice, obtained by mating *Hmox1*^+/–^ mice, survive to adulthood. Surviving *Hmox1*^–/–^ female mice were initially wrongly reported as infertile (Poss and Tonegawa, 1997; Yet et al., 1999; Tzima et al., 2009). Now we know that HO-1 knockout female are not infertile, but their gestations jeopardized due to the fact that the majority of their fetuses die *in utero* (Zenclussen et al., 2011). Hence, HO-1 emerges as a key molecule that regulates not one, but many, steps of female reproduction.

## HO-1 CONTRIBUTES TO OVULATION AND FERTILIZATION

The requirement of iron in adult life is around 4–5 mg daily (Andrews and Schmidt, 2007). The most common form is the reduced ferrous Fe^2+^ iron complexed with the two α-globin and two β-globin chains of heme to form hemoglobin. When red blood cells (RBCs) are lysed, iron is usually oxidized and heme no longer binds to hemoglobin, and is released as free heme. If left uncontrolled, free heme and oxidized iron can be harmful; both possessing pro-inflammatory, pro-oxidant, and tissue damaging properties (Soares and Bach, 2009). Free heme can be catabolized by HO-1. Insufficient iron uptake is related to subfertility. Women who consumed iron supplements had a significantly lower risk of ovulatory infertility than women who did not use iron supplements. Total non-heme iron intake, primarily consumed as multivitamins and iron supplements, was inversely associated with the risk of infertility. However, iron intake was unrelated to ovulatory infertility in multivariable-adjusted analyses (Chavarro et al., 2006). In rabbits, Lindahl et al. (1984) investigated the effect of ovulation on heme metabolism. HO activity, measured as total excretion of endogenous CO, was not increased after administration of any precursor for heme synthesis. However, CO increased 34% during the post-ovulation period. Because the increase in heme turnover after stimulation was small and may be unaccompanied by a contemporary increase in bilirubin/CO production, it was concluded that this increase in CO essentially was due to an increased destruction of circulating RBCs in the rabbit (Lindahl et al., 1984). For this step, elevated amounts of HO-1 are needed. Recently, we performed a mouse study designed to elucidate whether HO-1 is required for the process of oocyte production (ovulation) and subsequent corpus luteum (CL) formation. Thus, we documented the number of oocytes produced by *Hmox1* null mice compared to *Hmox1* wild-type (Wt) mice after pregnant mare serum gonadotropin (PMSG) plus human chorionic gonadotropin (hCG) hormonal stimulation. We observed that *Hmox1* null females produced significantly lower numbers of oocytes compared to *Hmox1* Wt mice in response to hormonal stimulation, clearly supporting a role for HO-1 in the process of ovulation (Zenclussen et al., 2012). We also analyzed how effectively these oocytes could be fertilized despite less oocytes were retrieved after hormonal stimulation. The fertilization rate of *Hmox1* null oocytes with sperm from knockout males was dramatically reduced as compared to Wt oocytes fertilized by Wt sperm. As sperm from *Hmox1*^+/+^ and *Hmox1*^–/–^ are equally fertile, it is clear that HO-1 expression in the oocytes modifies the ability of oocytes to be fertilized (Zenclussen et al., 2012). The total number of follicles was similar in both groups and the follicle composition was also comparable (Zenclussen et al., 2012). The number of CL was, however, significantly lower in *Hmox1* null than Wt mice, this being consistent with the lower number of ovulated oocytes from HO-1-deficient mice. Augmented apoptosis in the ovary of *Hmox1* null animals explained the lower number of CL (Zenclussen et al., 2012). After ovulation, immune cells, mostly macrophages and eosinophils, are located in the luteinizing theca area in the developing CL. This highlights the inflammatory nature of this process. The presence of HO-1 may represent one important counteracting mechanism to inflammation. In fact, this enzyme may confer cytoprotection during the inflammatory process of follicular rupture (Richards et al., 2002). Expression of HO-1 would then be required to prevent the cytotoxic effects of accumulated free heme. We proposed that the lack of HO-1 causes mature follicles to die as they are no longer protected from massive inflammation; hence, they will not ovulate (Zenclussen et al., 2012). Hence, HO-1 supports ovulation and the maintenance of the CL. Blastocyst attachment to the pregnant uterus occurs in a narrow period of time after fertilization called the “implantation window,” which is finely regulated by hormones released by the CL (van Mourik et al., 2009). As the proper on-time attachment of the blastocysts to the uterine wall has profound implications on posterior implantation and placentation (Song et al., 2002), the lack of HO-1 negatively impacts pregnancy outcomes as depicted by the lower number of and/or non-functional CL in mice whose HO-1 was ablated (Zenclussen et al., 2012).

The expression of HO-1 during the cycle seems to be regulated by hormones. It was reported that variations in HO-1 expression in the uterus correlate with changes in hormones at the four phases of the estrus cycle (Zenclussen et al., 2014). Interestingly, HO-1 protein expression is highest when female mice are receptive. *In vitro*, a combination of progesterone and estradiol can stimulate the expression of HO-1 in AN3 uterine cells (Zenclussen et al., 2014). Thus, hormones positively modulate HO-1 expression during receptivity, a crucial process for later implantation of the blastocyst.

## HO-1 CONTRIBUTES TO IMPLANTATION

Implantation is the earliest stage of pregnancy and is initiated when the blastocyst adheres to the wall of the uterus. Different processes take place during implantation. First, during the so-called “hatching,” the blastocyst gets rid of its zona pellucida. Immediately after, apposition takes place, which generates the very first connection between the blastocyst and the endometrium. In the following step, the adhesion is a much stronger attachment of the blastocyst to the endometrium. The first trophoblast begins penetrating the endometrium and this is, as mentioned before, a very aggressive and inflammatory process. The communication between the blastocyst and the endometrium at this stage is very intense. The blastocyst itself signals to the endometrium to adapt to its presence and is therefore the premise for correct invasion of the blastocyst into the endometrium and subsequent remodeling of tissues and spiral arteries.

The study of implantation mechanisms in the human is very difficult even though there are very elegant *in vitro* models. In Carlos Simón’s laboratory, a heterologous implantation model is used to study the ability of day 2 mouse embryos to attach to human endometrial epithelial cells (Garrido-Gomez et al., 2012; Vilella et al., 2013). Implantation in the mouse is very similar to humans. To understand the participation of HO-1 in implantation, we designed an *in vitro* mouse model to follow the hatching and invasion of the murine blastocyst into UECs grown in a stromal cell layer (Zenclussen et al., 2011). For this, we isolated and grew UECs in a layer of homologous stromal cells derived from either *Hmox1*-deficient or -sufficient animals at 3.5 day of pregnancy. From the same animals, we obtained blastocysts that were placed onto the UECs. With this model, we were able to observe that *Hmox1*^+/+^ blastocysts attached significantly faster than *Hmox1*^+/–^ or *Hmox1*^–/–^ blastocysts. *Hmox1*^–/–^ blastocysts failed to attach at all to UECs, which was not the case for *Hmox1*^+/+^, which were all attached at 72 h (Zenclussen et al., 2011). Evidently, HO-1 is expressed at the fetomaternal interface as early as at blastocyst implantation (Zenclussen et al., 2011). While not absolutely essential for implantation to occur, HO-1 supports timely blastocyst attachment to UECs *in vitro* (Zenclussen et al., 2011). Delayed implantation is known to have a profound impact on placentation and fetal growth (Song et al., 2002). *In vivo*, we could confirm that the size areas of whole *Hmox1*^+/–^ and *Hmox1*^–/–^ fetoplacental units were significantly smaller in contrast to whole *Hmox1*^+/+^ fetoplacental units already at gestational age day 8 (Linzke et al., 2014). This clearly means that the retarded attachment of the blastocyst to the UECs has serious consequences on the dynamics of implantation and thus on fetal growth. Chen et al. (2005) have identified genes that are expressed at a high level in hatched human blastocysts compared to pre-hatched blastocysts. Very interestingly, these hatching-specific genes were expressed at lower levels in the delayed growth embryos. Among the identified genes, HO-1 attracted our interest (Chen et al., 2005). Thus, it seems that also in humans, HO-1 is a crucial factor defining implantation success.

## HO-1 CONTRIBUTES TO PLACENTATION

It was reported by many research groups including ours that human invasive trophoblasts express HO-1 (Acevedo and Ahmed, 1998; Ahmed et al., 2000; Lyall et al., 2000). Murine invasive trophoblasts have typical giant cells (GC) morphological features, namely large-sized polyploid cells. GCs are the first cells from the ectoplacental clone to emerge and are critically involved in placentation. To study how HO-1 expression contributes to trophoblast physiology, we tested *in vitro* whether HO-1 is required for trophoblast proliferation or modulation of trophoblastic stem cell differentiation into GCs. For this, we employed the trophoblastic rat stem cell line Rcho-1 that can be induced to differentiate to GCs under determined conditions (Faria and Soares, 1991). Inhibition of HO-1 was achieved by the application of zinc protoporphyrin IX (ZnPP) and controls were treated with cobalt protoporphyrin IX (CoPP) that did not modify HO-1 expression. We observed that HO-1 inhibition reduced trophoblastic stem cell viability drastically (Zenclussen et al., 2011). We next differentiated stem cells into GCs and observed no changes in HO-1 expression during this process. The inhibition of HO-1 activity by ZnPP, however, suppressed trophoblastic stem cell differentiation into GCs, an effect not observed using CoPP. Thus, trophoblastic stem cells that express HO-1 can differentiate into GCs while cells lacking HO-1 cannot (Zenclussen et al., 2011). Hence, HO activity regulates both, trophoblast survival and differentiation into a mature phenotype, hence contributing importantly to placentation. Similarly, inhibition of HO-1 expression using siRNA inhibited viability of human primary trophoblasts isolated from first trimester pregnancies (Zenclussen et al., 2011).

Newby et al. (2005) analyzed HO-1 expression in human trophoblast cell cultures following exposure to different oxygen environments. They observed that HO-1 expression was stronger in cytotrophoblasts than in syncytiotrophoblasts. There was no difference in HO-1 expression in cytotrophoblasts transferred to low oxygen culture conditions. However, exposure of syncytiotrophoblast cultures to hypoxia for 12 h resulted in a significant reduction in HO-1 (42). Contradicting these results, Appleton et al. (2003b) tested HO-1 protein expression and enzymatic function in immortalized HTR-8/SVneo first-trimester trophoblast cells and explants of normal human chorionic villi from term placentas. They reported that HO-1 protein content was not affected by changes in oxygenation. They used 24-h exposures to 1, 5, or 20% oxygen (Appleton et al., 2003b). The same authors reported later that it is the combination of low oxygen and low glucose concentrations that decreased the HO-1 protein (Appleton et al., 2003a). Thus, it seems that in mice and humans, HO-1 is expressed in trophoblasts and plays an important regulatory role in its physiology.

The final confirmation that HO-1 is important for placentation was provided by mouse studies performed by Zhao et al. (2009) and our group. First, Zhao et al. (2009) showed that when *Hmox1*^+/–^ mice were cross-bred (FvB background), placentas and fetuses from *Hmox1*^+/–^ cross-breedings were altogether smaller and lighter than those from Wt cross-breedings. Shortly after, we analyzed the histopathology of placentas obtained from *Hmox1*^+/+^ × *Hmox1*^+/+^, *Hmox1*^+/–^ × *Hmox1*^+/–^, and *Hmox1*^+/–^ × *Hmox1*^–/–^ combinations (BALB/c background) after genotyping their respective embryos. We observed that the number of GCs in placentas totally or partially deficient in *Hmox1* was significantly reduced as compared to Wt (*Hmox1*^+/+^) placentas. In addition, *Hmox1*^+/–^ and *Hmox1*^–/–^ placentas had enlarged labyrinth areas and reduced or absent junctional areas with detectable morphological abnormalities, confirming improper placentation. They further presented areas of fibrosis and hemorrhage (Zenclussen et al., 2011). Thus, deletion of the *Hmox1* allele leads to abnormal placentation and likely to insufficient nutrient and oxygen supply to the fetus. When mating *Hmox1*^+/–^ with *Hmox1*^+/–^, there were no viable *Hmox1*^–/–^ fetuses detectable at day 12; while *Hmox1*^+/–^ were significantly much smaller than *Hmox1*^+/+^ ones. Impressively, most fetuses from *Hmox1*^–/–^ × *Hmox1*^–/–^ combinations were non-viable at day 14. These observations provide conclusive evidence that deletion of the *Hmox1* allele has major pathological consequences during pregnancy leading to abnormal placentation, intrauterine growth restriction (IUGR), and eventually to fetal loss (Zenclussen et al., 2011). Remarkably, numerous studies have reported that HO-1 is diminished in cases of pre-eclampsia and IUGR (Ahmed et al., 2000; Lyall et al., 2000; Zenclussen et al., 2003).

## HO-1 CONTRIBUTES TO SPIRAL ARTERY DEVELOPMENT

Pregnancy success very much depends on adequate placentation to ensure proper oxygen and nutrient supplies to the developing fetus. Uteroplacental blood flow increases dramatically during gestation, this being facilitated by the growth and remodeling of the maternal uterine spiral artery (SA) system. It is widely demonstrated that insufficient uterine vascular remodeling leads to pregnancy-associated pathologies like IUGR and pre-eclampsia (Bewley et al., 1991; Bernstein et al., 1998). We recently showed that the expression of angiogenic factors was diminished at the fetomaternal interface of *Hmox1*^–/–^ and *Hmox1*^+/–^ implantations when compared to *Hmox1*^+/+^ implantations at gestational days 8, 10, and 12. This was associated with lower numbers of uNK cells, which is discussed below. These cells are necessary for the correct SA remodeling. Therefore, we studied whether HO-1 contributed not only to diminished expression of angiogenic factors; but also, and more relevantly to disturbances in SA remodeling. We found that at days 10 and 12 of pregnancy, SA remodeling was dramatically impaired in *Hmox1*^+/–^ and *Hmox1*^–/–^ implantation sites when compared to *Hmox1*^+/+^ controls (Linzke et al., 2014). The ratio of wall to lumen diameter of the SA was significantly increased in *Hmox1*^+/–^ and *Hmox1*^–/–^ implantations compared to Wt implantations (Linzke et al., 2014). A thicker wall and thus, a smaller lumen, means a restricted blood supply to the fetus, which can have fatal consequences on its growth. In fact, we observed that *Hmox1*^+/–^ and *Hmox1*^–/–^ concepti were either severely growth retarded or suffered intrauterine deaths (Zenclussen et al., 2011; Linzke et al., 2014). Wong and colleagues also showed that a partial deficiency in HO-1 resulted in smaller babies (Zhao et al., 2009). To confirm that the insufficient HO-1 levels are directly related to the inadequate SA remodeling, we treated *Hmox1*-deficient animals with the HO metabolite CO. CO could not only normalize the number of uNKs and the expression of angiogenic factors, but impressively restored SA remodeling (Linzke et al., 2014). Thus, the expression of HO-1 at the fetomaternal interface is indispensable for optimal SA remodeling that, in turn, ensures the timely growth of the fetus. This is in agreement with data from human pregnancies. Several authors showed that HO-1 is diminished at the fetomaternal interface of patients with normotensive IUGR or pre-eclampsia when compared to age-matched controls (Ahmed et al., 2000; Lyall et al., 2000; Zenclussen et al., 2003). In these particular pregnancy complications, a suboptimal SA remodeling was described (Bernstein et al., 1998).

## HO-1 CONTRIBUTES TO BLOOD PRESSURE REGULATION DURING PREGNANCY

If SA remodeling is not optimal, the maternal body will attempt to supply the fetus with more blood, which can lead to hypertension. It therefore can be expected that a suboptimal SA remodeling due to HO-1 deficiency is associated with increased blood pressure. We recently showed that HO-1 deficiency altered the number of uNKs, their angiogenic factors secreted, and negatively interfered with SA remodeling, thus resulting in IUGR. But most importantly, we could show that HO-1-deficient females developed hypertension beginning at day 14 of pregnancy (Linzke et al., 2014). Hypertension is thought to be a consequence of an insufficient blood supply to the fetus following impaired SA remodeling (Robson et al., 2012). Thus, animals deficient in HO-1 resemble a model of naturally-occurring pre-eclampsia (Linzke et al., 2014). These findings are complemented by previous studies from Zhao et al. (2009) who showed an increase in diastolic blood pressure in pregnant HO-1-deficient mice. In the same line of evidence, George et al. (2013) showed that the treatment of pregnant rats with the HO-1 inhibitor tin mesoporphyrin (SnMP) provoked gestational hypertension. In response to SnMP treatment, maternal arterial pressure not only increased; but also, placental VEGF was decreased (George et al., 2013). In a rat model in which hypertension was induced by continuous infusion of recombinant sFlt-1, George et al. (2011) could show that the HO-1 induction after application of CoPPIX helped normalize blood pressure. The mechanisms behind this were not investigated and are unclear, especially because neither our group nor George and colleagues could find a correlation between HO-1 and sFlt-1 (George et al., 2013; Linzke et al., 2014). However, at least one other paper reported that *in vitro*, HO-1 and/or CO downregulated sFlt-1 production (Cudmore et al., 2007). The role of HO-1 in preventing gestational hypertension was confirmed after observing that CO application during days 3–8 could compensate for HO-1 deficiency and reversed the development of hypertension and IUGR in a mouse model (Linzke et al., 2014). Cigarette smoking during pregnancy is obviously a non-healthy habit that is associated with adverse maternal, placental, and fetal effects, e.g., preterm-delivery, abnormal placentation and small-for-gestational age (SGA) fetuses (Bainbridge et al., 2005). However, women smoking cigarettes during pregnancy have a reduced risk developing pre-eclampsia (Conde-Agudelo et al., 1999; England and Zhang, 2007). One mechanism through which cigarette smoke may decrease the risk of developing pre-eclampsia in pregnancy is the essential element CO (Zhai et al., 2012), while the other cigarette components may account for the negative effects. This warrants further investigation. It is important to remark that both, dose and time of exposure to CO are very important. To achieve positive effects on gestational hypertension was achieved at very low doses of 50 ppm during the process of periimplantation (days 3–8 of pregnancy). Higher doses were detrimental while shorter time exposures had no relevant effect (El-Mousleh et al., 2012). We showed that CO application during early mouse pregnancy could actually prevent hypertension and IUGR (Linzke et al., 2014). CO therapy should be explored in more detail, especially after the recent report on decreased risk of pre-eclampsia after maternal exposure to moderate ambient CO (Zhai et al., 2012). In animals that spontaneously develop pre-eclampsia, hypertension was first detectable at day 14, while CO application prevented this only if applied from day 3 to day 8 of pregnancy (Linzke et al., 2014). It is clear then that the events leading to pre-eclampsia development are triggered and can be corrected beginning as early as day 3. In humans, a shallow invasion of the trophoblast at week 14, long before pre-eclamptic symptoms arise, is responsible for the pathophysiology of this complex disease (Redman and Sargent, 2005). We previously proposed that the excess of free heme in *Hmox1*^+/–^ animals contributes in part to gestational hypertension (Zenclussen et al., 2011). These animals are not hypertensive before or at the beginning of pregnancy. However, as pregnancy advances and the circulating blood volume increases, low levels of HO-1 are not enough to remove the excessive free heme, known to be cytotoxic. It was shown that pre-eclamptic placentas that express less HO-1 also had a reduced phosphorylation of phosphoinositide kinase (PI3K)/Akt, and this correlated with the elevated levels of circulating sEng. In trophoblasts, knockdown of Akt prevented HO-1-mediated inhibition of sEng release and reduced HO-1 expression. Accordingly, non-pregnant Hmo1 null mice had reduced phosphorylated Akt in their organs and overexpression of Akt(myr) failed to suppress the elevated levels of sEng detected in HO-1 null mice, indicating that HO-1 is required for the Akt-mediated inhibition of sEng (Cudmore et al., 2012). Thus, it seems possible that the loss of PI3K/Akt and/or HO activity promotes sEng release; positive manipulation of these pathways may offer a strategy to circumvent endothelial dysfunction.

## HO-1 PARTICIPATION DURING PREGNANCIES JEOPARDIZED BY INFECTIONS

Because of its central role in preventing inflammation, HO-1 may be also important in pregnancies that are in danger because of the presence of pathogens. Recently, Penha-Goncalves et al. (2014) proposed an important role for HO-1 during gestational malaria. In fact, plasmodium infection during gestation is related to severe clinical manifestations including abortion and stillbirth. In a mice model of severe placental malaria (after infection with *Plasmodium berghei*), both mothers and fetuses often succumb to infection before or immediately after delivery (Mineo et al., 2013). *P. berghei*-infected erythrocytes accumulate in the placenta, where they are then phagocytized by trophoblasts (de Moraes et al., 2013; Lima et al., 2014). Inside the phagosomes, disruption of the RBCs provokes the release of free heme, which has to be catabolized by HO-1. Results of Penha-Goncalves et al.’s (2014) studies suggest that free heme-derived iron overload in infected trophoblasts leads to fetal death. Only placentas with no visible iron accumulation could support the normal growth of fetuses. Thus, increased HO activity would protect fetuses from injury. Accordingly, Woudwyk et al. (2012) showed that pregnant BALB/c female mice that have been infected with *Tritrichomonas foetus* had strongly decreased levels of HO-1 in those concepti that died immediately after infections. In a study by Tachibana et al. (2011), it could be showed that *Listeria monocytogenes* infection in trophoblast GCs decreased HO-1 and Bcl-XL. Their upregulation, however, inhibited cell death induced by the infection. Accordingly, HO-1 and Bcl-XL expression levels were decreased after *L. monocytogenes* infection of pregnant mice. Treatment with CoPP inhibited infectious abortion (Tachibana et al., 2011). This goes hand in hand with the protective effects of both HO-1-based gene therapy and CoPPIX application in pregnancies (Sollwedel et al., 2005; Zenclussen et al., 2006). Similarly, in a model of *Brucella abortus*, it was shown that the expression of HO-1 in the placenta was decreased by *B. abortus* infection (Tachibana et al., 2008). Again, CoPPIX was able to inhibit abortions due to the bacterial infection (Tachibana et al., 2008). Thus, HO-1 emerges as a protective mechanism in placental cells, in charge of keeping the tissue under surveillance in order to avoid inflammatory consequences that can lead to embryonic or fetal death.

## MECHANISMS BEHIND THE PROTECTIVE EFFECTS OF HO-1 IN PREGNANCY

In recent years, key evidences emerged showing that HO-1 is essential for many steps of pregnancy. It is therefore of vital importance to understand the mechanisms underlying the protective effects of HO-1. The mechanisms may vary depending on the reproductive phase HO-1 with which is involved. The microenvironment where HO-1 action is required may also have a significant influence on the mechanisms activated. The best study mechanism underlying HO-1 effects that may account for several of its positive gestational effects is the release of CO. This accounts not only for reproductive processes (Cudmore et al., 2007; Zenclussen et al., 2011; El-Mousleh et al., 2012; Linzke et al., 2014); but also, for many inflammatory processes (Gozzelino et al., 2010; Wegiel et al., 2014a,b). Already at the trophoblast stage, we observed that the application of CO could overcome the negative effects of ZnPP treatment to trophoblast stem cells and enable their proliferation and further conversion into GCs (Zenclussen et al., 2011). Similarly, the addition of CO to cultured human trophoblasts from patients with pre-eclampsia diminished their ability to secrete sFlt-1 (Cudmore et al., 2007). Free heme *per se* is sufficient to precipitate intrauterine fetal deaths in Wt *Hmox1*^+/+^ mice, and this is independent of the maternal adaptive immune system (Zenclussen et al., 2011). It can, however, be suppressed by the administration of low doses of CO. Indeed, exogenous CO applied between pre-implantation until placentation prevents fetal loss in *Hmox1*^+/–^ females mated with *Hmox1*^+/–^ males as compared to air-treated controls. More importantly, CO can rescue *Hmox1*^–/–^ fetuses that die otherwise *in utero* (Zenclussen et al., 2011). The protective effect of CO was associated primarily with the normalization of placenta morphology (Zenclussen et al., 2011). In further studies, we showed that the prevention of fetal deaths by CO might be due to both the prevention of free heme deposition and the augmentation in the uNK cell numbers (Linzke et al., 2014). uNK cells contribute to the invasion of trophoblast cells in early pregnancy. Insufficient uNK cell activation in early pregnancy leads to an imbalance in vascular formation and this may cause the onset of pre-eclampsia in late pregnancy. HO-1-deficient mice have reduced uNK cell numbers and develop hypertension at mid- to late pregnancy (Linzke et al., 2014). From our published results, we propose that the absence of HO-1 interferes with the phenotype of macrophages (M1 rather than M2) that in turns provokes less IL-15 secretion, thereby reducing uNK cell numbers (Linzke et al., 2014). HO-1 deficiency resulted not only in fewer uNKs; but also, in shallow SA remodeling and IUGR (Linzke et al., 2014). The administration of CO from gestational day 3 until day 8 provoked a significant increase in the number of uNKs that was independent of IL-15 at the implantation sites (Linzke et al., 2014). We confirmed that CO acts directly on uNKs inducing their *in situ* proliferation (Linzke et al., 2014). As in other models (Dulak et al., 2002), CO could positively influence angiogenesis by stimulating the production of VEGF and PGF, positively influencing the remodeling of the maternal uterine vascular system (Linzke et al., 2014). It is known that diminished signaling of VEGF and PGF in the placenta is aligned with IUGR and pre-eclampsia in humans (Maynard et al., 2005). Accordingly, there was impaired VEGF, PGF, and IFN-γ mRNA expression at the fetomaternal interface of *Hmox1*^+/–^ and *Hmox1*^–/–^ implantation sites compared to Wt controls. CO application to pregnant *Hmox1*^+/–^ females not only favored the uNK cell proliferation; but also, enhanced the expression of VEGF, PGF, and IFN-γ that accompanied SA reshaping (Linzke et al., 2014). Thus, CO not only protects from an excess of free heme; but also, positively stimulates uNK proliferation and thus angiogenesis, both of great importance in the prevention of pre-eclampsia and IUGR development.

Clark et al. (1998) reported that the placenta secretes a soluble factor named sFlt-1 that can bind VEGF, thus being its antagonist. In pre-eclampsia, sFlt-1 is elevated and has since been considered a marker for pre-eclampsia (Vuorela et al., 2000; Buhimschi et al., 2005). In Wt mice, neutralization of VEGF-induced proteinuria (Sugimoto et al., 2003) and sFlt-1 gene transfer provoked pre-eclampsia-like signs (Maynard et al., 2003; Lu et al., 2007). Cudmore et al. (2007) showed that, *in vitro*, the overexpression of HO-1 in endothelial cells by using a retrovirus-inhibited sFlt-1 release, whereas HO-1 inhibition potentiated sFlt-1 production from endothelial cells and placental villous explants. Cudmore et al. (2012) also reported that non-pregnant mice lacking HO-1 produced higher levels of sFlt-1 than Wt mice. However, we were not able to find any differences in sFlt-1 levels among *Hmox1*^+/+^, *Hmox1*^+/–^, and *Hmox1*^–/–^ female mice during pregnancy (Vilella et al., 2013). It was reported that CO is able to decrease sFlt-1 release *in vitro* (Cudmore et al., 2012). *In vivo*, CO application has no effect on sFlt-1 levels regardless of the mouse *Hmox1* phenotype (Linzke et al., 2014). This was also true for s-Eng, another pre-eclampsia marker (Linzke et al., 2014). Similarly, placental sFlt-1 levels in pregnant rats were not affected by HO inhibition (George et al., 2011). There are other studies in which CO was able to diminish sFlt-1 levels, but this seems to be independent of HO-1. In a mouse model of pre-eclampsia-induced after sFlt-1 infusion, CO application ameloriated pre-eclampsia signs (Venditti et al., 2014). However, the doses employed in this study are very high (250 ppm) and even though the authors quote this dose as “low,” previous studies showed that at even lower doses (125 ppm), CO has toxic effects on both the mother and fetus (El-Mousleh et al., 2012). At 50 ppm, CO was able to ameliorate the rate of fetal deaths in the abortion-prone mouse combination CBA/J × DBA/2J and reduce both sFlt-1 and sEng levels (El-Mousleh et al., 2012).

## OUTLOOK: POTENTIAL OF HO-1 AS A THERAPEUTIC TARGET IN PREGNANCY COMPLICATIONS

CO might be used therapeutically to suppress early and recurrent onset of spontaneous abortions or fetal deaths associated with human HMOX1 polymorphisms (Otterbein et al., 2000) or to prevent IUGR in pre-eclampsia-associated with low HO-1 levels (Barber et al., 2001). We found that CO can also be used therapeutically to fully reverse IUGR and gestational hypertension when applied exogenously during late implantation and throughout placentation. This confirms the great potential of CO for pregnancy complications as it is already well known to be protective in a variety of pathologies (Otterbein et al., 2000; Pamplona et al., 2007; Motterlini and Otterbein, 2010). Pre-eclampsia, known to be caused by shallow trophoblast invasion, hence, abnormal placentation, has not only been linked to HO-1 deficiency, but its incidence is significantly lower in smokers (England et al., 2003; Khoury et al., 2004), and as discussed, CO has been proposed as the main reason for this. Recently, Ahmed proposed to use the cardiovascular drugs statins (3-hydroxy-3 methyl-glutaryl coenzyme-A reductase inhibitors) to stimulate HO-1 expression and inhibit sFlt-1 in pre-eclamptic patients (Ahmed, 2011). In mice, statins prevented cervical remodeling, myometrial contractions, and preterm labor in a LPS-induced model of preterm birth. Interestingly, the protective effects of statins in this model were associated with increased synthesis, expression, and activity of HO-1 in the myometrium and cervix. Co-administration of the HO-1 inhibitor tin protoporphyrin (SnPP) abrogated the protective effects of statins and preterm births could no longer been prevented (Gonzalez et al., 2014). These promising results show the potential of these drugs, but nevertheless careful studies are required before their universal use (Cleary et al., 2014). In rats, pravastatin ameloriated hypertension, oxidative stress, and angiogenic imbalance in a rat model of placental ischemia-induced hypertension (Bauer et al., 2013). Pravastatin further prevented the rise in circulating anti-angiogenic factors in a mouse model of pre-eclampsia. Statins may represent a novel approach to prevention of this devastating disease (Saad et al., 2014). Maternal therapy with pravastatin in mice with sFlt-1-induced pre-eclampsia could prevent alterations in postnatal growth and metabolic functions in the adult offspring of pre-eclamptic mice (McDonnold et al., 2014). Similarly, prenatal pravastatin treatment prevented impairment of fetal programming in mice (Carver et al., 2014). There is to date one case report from a patient with pre-eclampsia that was successfully treated with pravastatin (Lefkou et al., 2014). Because pravastatin and other statins have been shown to reverse various pathophysiologic pathways associated with pre-eclampsia, such as angiogenic imbalance, endothelial injury, inflammation, and oxidative stress, it is claimed to have favorable safety and pharmacokinetic profiles. National Institute of Child Health and Human Development Obstetric-Fetal Pharmacology Research Units Network has started a pilot trial to collect maternal/fetal safety data and to evaluate pravastatin pharmacokinetics when used as a prophylactic daily treatment in high-risk pregnant women (identifier NCT01717586, clinicaltrials.gov; Costantine et al., 2013). The future will show whether these drugs can help ameliorate pre-eclamptic symptoms and whether HO-1/CO are involved.

## SUMMARY

**FIGURE 1:**
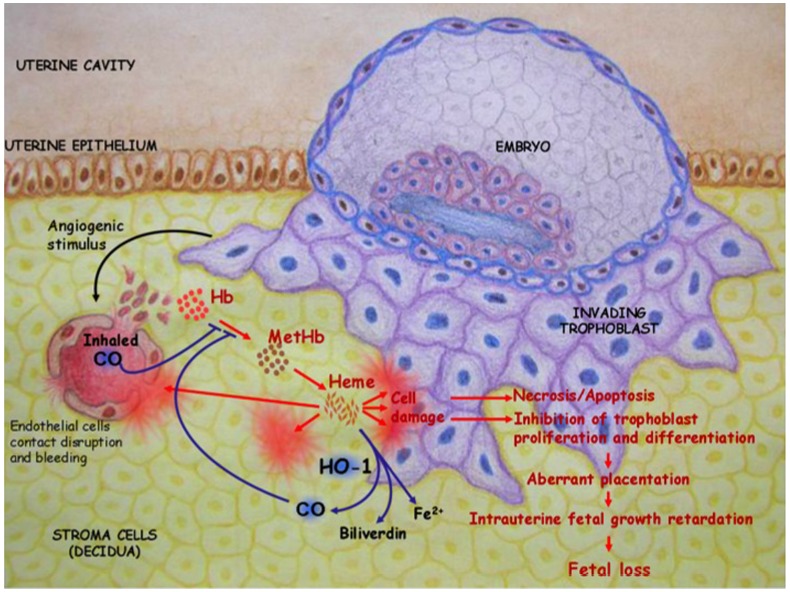
**HO-1 catabolizes free heme into biliverdin and releases CO.** This supports the implantation of the fertilized blastocyst. In the absence of HO-1, excess free heme provokes cell damage that in turn leads to necrosis and/or apoptosis. This impairs trophoblast functions, resulting in abnormal placentation, IUGR, and fetal deaths. These negative consequences of excess free heme could be prevented in a mouse model by inhalation of low doses of CO (50 ppm).

HO-1 is a unique regulator of reproductive processes in the female. As depicted in Figure 1, HO-1 supports the implantation of the fertilized blastocyst and is mediated by its metabolite CO, which avoids the accumulation of free heme. The on-time implantation in the presence of HO-1 is followed by a normal placentation, fetal growth, and well-being. In the absence of HO-1, excess free heme provokes cell damage and results in a cascade of events that finally leads to aberrant placentation, IUGR, and/or fetal loss. These negative consequences of excess free heme can be prevented by the administration of CO. HO-1 and its metabolite CO both have a great therapeutic potential in reproductive medicine and reproductive immunology. Further studies are required to fully understand their mechanisms of action and how to modulate their levels *in situ*.

### Conflict of Interest Statement

The authors declare that the research was conducted in the absence of any commercial or financial relationships that could be construed as a potential conflict of interest.
